# Supplementary material for: Hydrop enables droplet-based single-cell ATAC-seq and single-cell RNA-seq using dissolvable hydrogel beads
Source: eLife. 2022 Feb 23;11:e73971. doi: 10.7554/eLife.73971 (PMC8993220; doi:10.7554/eLife.73971)
Supplement: Figure 1—figure supplement 2—source data 1. — Beads were incubated in fluorescent probes complementary to basal acrydite primer or poly-A or ATAC capture site. Absolute size of 1 SD is added in mean diameter and mean intensity columns. [file elife-73971-fig1-figsupp2-data1.docx]

| **Method** | **Acrydite (uM)** | **Type** | **Stage** | **Probe** | **n** | **dmean (μm)** | **imean** |
| --- | --- | --- | --- | --- | --- | --- | --- |
| **Isothermal ext.** | **50** | RNA | Pre BC | Acrydite | 34 | 57.63 ± 1.68 | 102.03 ± 1.1 |
|  |  |  | Post BC | Acrydite | 43 | 61.44 ± 2.21 | 76.18 ± 14.3 |
|  |  |  | Post BC | PolyA | 40 | 59.6 ± 2.84 | 16.51 ± 5.85 |
| **Linear amp.** | **12** | RNA | Pre BC | Acrydite | 271 | 63.16 ± 2.4 | 60.42 ± 4.91 |
|  |  |  | Pre BC | Acrydite | 357 | 62.53 ± 2.59 | 52.3 ± 6.64 |
|  |  |  | Post BC | PolyA | 243 | 64.87 ± 2.73 | 51.95 ± 6.71 |
|  |  | ATAC | Post BC | Acrydite | 517 | 45.91 ± 1.3 | 44.82 ± 5.3 |
|  |  |  | Pre BC | ATAC | 351 | 45.96 ± 1.09 | 36.33 ± 5.9 |
