# Supplementary material for: Hydrop enables droplet-based single-cell ATAC-seq and single-cell RNA-seq using dissolvable hydrogel beads
Source: eLife. 2022 Feb 23;11:e73971. doi: 10.7554/eLife.73971 (PMC8993220; doi:10.7554/eLife.73971)
Supplement: Figure 1—figure supplement 3—source data 1. [file elife-73971-fig1-figsupp3-data1.docx]

|  | **n** | **Positive** | **Rate (%)** | **1 in …** |
| --- | --- | --- | --- | --- |
| **Round 1** | 570 | 7 | 1.23% | 81.4 |
| **Round 2** | 425 | 7 | 1.65% | 60.7 |
| **Round 3** | 669 | 5 | 0.75% | 133.8 |
| **Total** | 1664 | 19 | 1.14% | 87.6 |
