# Supplementary material for: Hydrop enables droplet-based single-cell ATAC-seq and single-cell RNA-seq using dissolvable hydrogel beads
Source: eLife. 2022 Feb 23;11:e73971. doi: 10.7554/eLife.73971 (PMC8993220; doi:10.7554/eLife.73971)
Supplement: Figure 1—figure supplement 4—source data 1. — Absolute size of 1 SD is added in mean diameter and mean intensity columns. [file elife-73971-fig1-figsupp4-data1.docx]

| **Time in DTT** | **n** | **dmean (μm)** | **imean** |
| --- | --- | --- | --- |
| **0 min.** | 108 | 47.77 ± 1.69 | 82.2 ± 2.63 |
| **15 min.** | 96 | 48.14 ± 1.48 | 81.98 ± 2.32 |
| **50 min.** | 112 | 46.57 ± 1.81 | 80.24 ± 3.22 |
