# Supplementary material for: Hydrop enables droplet-based single-cell ATAC-seq and single-cell RNA-seq using dissolvable hydrogel beads
Source: eLife. 2022 Feb 23;11:e73971. doi: 10.7554/eLife.73971 (PMC8993220; doi:10.7554/eLife.73971)
Supplement: Supplementary file 1. [file elife-73971-supp1.docx]

**Supplementary file to “HyDrop enables droplet based single-cell ATAC-seq and single-cell RNA-seq using dissolvable hydrogel beads”**

Florian V. De Rop^1,2^, Joy N. Ismail^1,2^, Carmen Bravo González-Blas^1,2^, Gert J. Hulselmans^1,2^, Christopher C. Flerin^1,2,4^, Jasper Janssens^1,2^, Koen Theunis^1,2,4^, Valerie M. Christiaens^1,2^, Jasper Wouters^1,2^, Gabriele Marcassa^1,3^, Joris de Wit^1,3^, Suresh Poovathingal^1,#^, and Stein Aerts^1,2,#^

^1^ VIB-KU Leuven Center for Brain & Disease Research

^2^ Laboratory of Computational Biology, Department of Human Genetics, KU Leuven

^3^ Laboratory of Synapse Biology, Department of Neurosciences, KU Leuven

^4^ Aligning Science Across Parkinson’s (ASAP) Collaborative Research Network, Chevy Chase, MD,= 20815.

^#^ Shared last author; correspondence to [suresh.poovathingal@kuleuven.be](mailto:suresh.poovathingal@kuleuven.be) and [stein.aerts@kuleuven.be](mailto:stein.aerts@kuleuven.be).

**Sequence on the unbarcoded hydrogel bead:**

**/5Acryd//iThioMC6-D/TTTTTTTTAATACGACTCACTATAGGGAAGCAGTGGTATCAACGCAGAGTAC**

**Sequences in red denote one of 96 possible sub-barcodes.**

**Extension 1:**

**/5Acryd//iThioMC6-D/TTTTTTTTAATACGACTCACTATAGGGAAGCAGTGGTATCAACGCAGAGTACTTCCTGTGAGCAGCTACTGC**

**GCGTCTCATGAAGGACACTCGTCGATGACG**

**Extension 2:**

**/5Acryd//iThioMC6-D/TTTTTTTTAATACGACTCACTATAGGGAAGCAGTGGTATCAACGCAGAGTACTTCCTGTGAGCAGCTACTGCTCGGACTTATCGAGTACCCT**

**GTCGATGACGAGCCTGAATAGCTCATGGGA**

**Extension 3 (HyDrop-RNA):**

**/5Acryd//iThioMC6-D/TTTTTTTTAATACGACTCACTATAGGGAAGCAGTGGTATCAACGCAGAGTACTTCCTGTGAGCAGCTACTGCTCGGACTTATCGAGTACCCTGGCTGAATTANNNNNNNNNTTTTTTTTTTTTTTTTTTTTTTTTT**

**GCTCATGGGACCGACTTAATNNNNNNNNNAAAAAAAAAAAAAAAAAAAAAAAAA**

**Final primer sequence on bead for HyDrop-RNA:**

**PCR handle BC1 BC2 BC3**

**/5Acryd//iThioMC6-D/TTTTTTTTAATACGACTCACTATAGGGAAGCAGTGGTATCAACGCAGAGTACTTCCTGTGAGCAGCTACTGCTCGGACTTATCGAGTACCCTGGCTGAATTANNNNNNNNNTTTTTTTTTTTTTTTTTTTTTTTTT 136 bp**

**Extension 3 (HyDrop-ATAC):**

**/5Acryd//iThioMC6-D/TTTTTTTTAATACGACTCACTATAGGGAAGCAGTGGTATCAACGCAGAGTACTTCCTGTGAGCAGCTACTGCTCGGACTTATCGAGTACCCTGGCTGAATTAGTCTCGTGGGCTCGG**

**GCTCATGGGACCGACTTAATCAGAGCACCCGAGCC**

**Final primer sequence on bead for HyDrop-ATAC:**

**PCR handle BC1 BC2 BC3**

**/5Acryd//iThioMC6-D/TTTTTTTTAATACGACTCACTATAGGGAAGCAGTGGTATCAACGCAGAGTACTTCCTGTGAGCAGCTACTGCTCGGACTTATCGAGTACCCTGGCTGAATTAGTCTCGTGGGCTCGG 117 bp**
