# Supplementary material for: Hydrop enables droplet-based single-cell ATAC-seq and single-cell RNA-seq using dissolvable hydrogel beads
Source: eLife. 2022 Feb 23;11:e73971. doi: 10.7554/eLife.73971 (PMC8993220; doi:10.7554/eLife.73971)
Supplement: Supplementary file 2. [file elife-73971-supp2.docx]

**Supplementary file to “HyDrop enables droplet based single-cell ATAC-seq and single-cell RNA-seq using dissolvable hydrogel beads”**

Florian V. De Rop^1,2^, Joy N. Ismail^1,2^, Carmen Bravo González-Blas^1,2^, Gert J. Hulselmans^1,2^, Christopher C. Flerin^1,2,4^, Jasper Janssens^1,2^, Koen Theunis^1,2,4^, Valerie M. Christiaens^1,2^, Jasper Wouters^1,2^, Gabriele Marcassa^1,3^, Joris de Wit^1,3^, Suresh Poovathingal^1,#^, and Stein Aerts^1,2,#^

^1^ VIB-KU Leuven Center for Brain & Disease Research

^2^ Laboratory of Computational Biology, Department of Human Genetics, KU Leuven

^3^ Laboratory of Synapse Biology, Department of Neurosciences, KU Leuven

^4^ Aligning Science Across Parkinson’s (ASAP) Collaborative Research Network, Chevy Chase, MD,= 20815.

^#^ Shared last author; correspondence to [suresh.poovathingal@kuleuven.be](mailto:suresh.poovathingal@kuleuven.be) and [stein.aerts@kuleuven.be](mailto:stein.aerts@kuleuven.be).

**HyDrop-ATAC**

**ATAC Reaction in bulk**

**5-GTCTCGTGGGCTCGGAGATGTGTATAAGAGACAGXXXXXXXXXXXX...XXX CTGTCTCTTATACACATCT-3**

**3-TCTACACATATTCTCTGTC XXX...XXXXXXXXXXXXGACAGAGAATATGTGTAGACTGCGACGGCTGCT-5**

**Linear PCR amplification in Emulsion**

**Gap Fill step**

**5-GTCTCGTGGGCTCGGAGATGTGTATAAGAGACAGXXXXXXXXXXXX...XXXXXXXXXXXXCTGTCTCTTATACACATCTGACGCTGCCGACGA**

**3-CAGAGCACCCGAGCCTCTACACATATTCTCTGTCXXXXXXXXXXXX...XXXXXXXXXXXXGACAGAGAATATGTGTAGACTGCGACGGCTGCT-5**

**Barcode captures fragment**

**5-TTTTTTTTAATACGACTCACTATAGGGAAGCAGTGGTATCAACGCAGAGTACTTCCTGTGAGCAGCTACTGCTCGGACTTATCGAGTACCCTGGCTGAATTAGTCTCGTGGGCTCGG-3**

**3-CAGAGCACCCGAGCCTCTACACATATTCTCTGTCXXXXXXXXXXXX...XXXXXXXXXXXXGACAGAGAATATGTGTAGACTGCGACGGCTGCT-5**

**Linear PCR**

**5-TTTTTTTTAATACGACTCACTATAGGGAAGCAGTGGTATCAACGCAGAGTACTTCCTGTGAGCAGCTACTGCTCGGACTTATCGAGTACCCTGGCTGAATTAGTCTCGTGGGCTCGGAGATGTGTATAAGAGACAGXXXXXXXXXXXX...XXXXXXXXXXXXCTGTCTCTTATACACATCTGACGCTGCCGACGA-3**

**3-CAGAGCACCCGAGCCTCTACACATATTCTCTGTCXXXXXXXXXXXX...XXXXXXXXXXXXGACAGAGAATATGTGTAGACTGCGACGGCTGCT-5**

**5-TTTTTTTTAATACGACTCACTATAGGGAAGCAGTGGTATCAACGCAGAGTACTTCCTGTGAGCAGCTACTGCTCGGACTTATCGAGTACCCTGGCTGAATTAGTCTCGTGGGCTCGGAGATGTGTATAAGAGACAGXXXXXXXXXXXX...XXXXXXXXXXXXCTGTCTCTTATACACATCTGACGCTGCCGACGA-3**

**5-TTTTTTTTAATACGACTCACTATAGGGAAGCAGTGGTATCAACGCAGAGTACTTCCTGTGAGCAGCTACTGCTCGGACTTATCGAGTACCCTGGCTGAATTAGTCTCGTGGGCTCGGAGATGTGTATAAGAGACAGXXXXXXXXXXXX...XXXXXXXXXXXXCTGTCTCTTATACACATCTGACGCTGCCGACGA-3**

**:**

**:**

**5-TTTTTTTTAATACGACTCACTATAGGGAAGCAGTGGTATCAACGCAGAGTACTTCCTGTGAGCAGCTACTGCTCGGACTTATCGAGTACCCTGGCTGAATTAGTCTCGTGGGCTCGGAGATGTGTATAAGAGACAGXXXXXXXXXXXX...XXXXXXXXXXXXCTGTCTCTTATACACATCTGACGCTGCCGACGA-3**

**PCR amplification in bulk (post emulsion breakage)**

**Sequencing library preperation**

**5-TTTTTTTTAATACGACTCACTATAGGGAAGCAGTGGTATCAACGCAGAGTACTTCCTGTGAGCAGCTACTGCTCGGACTTATCGAGTACCCTGGCTGAATTAGTCTCGTGGGCTCGGAGATGTGTATAAGAGACAGXXXXXXXXXXXX...XXXXXXXXXXXXCTGTCTCTTATACACATCTGACGCTGCCGACGA-3**

**3-** **GTGTAGACTGCGACGGCTGCTGCGAGGTGCTCACATCTAGAGCCACCAGCGGCATAGTAA-5**

**5-CAAGCAGAAGACGGCATACGAGATCGCTCAGTTCCTGTCCGC\**

**\GGAAGCAGTGGTATCAACGCAGAGTAC-3**

**/CCTTCGTCACCATAGTTGCGTCTCATGAAGGACACTCGTCGATGACGAGCCTGAATAGCTCATGGGACCGACTTAATCAGAGCACCCGAGCCTCTACACATATTCTCTGTCXXXXXXXXXXXX...XXXXXXXXXXXXGACAGAGAATATGTGTAGACTGCGACGGCTGCT-5**

**3-AAAAAAAATTATGCTGAGTGATATC/**

**5-CAAGCAGAAGACGGCATACGAGATCGCTCAGTTCCTGTCCGCGGAAGCAGTGGTATCAACGCAGAGTACTTCCTGTGAGCAGCTACTGCTCGGACTTATCGAGTACCCTGGCTGAATTAGTCTCGTGGGCTCGGAGATGTGTATAAGAGACAGXXXXXXXXXXXX...XXXXXXXXXXXXCTGTCTCTTATACACATCTGACGCTGCCGACGACGCTCCACGAGTGTAGATCTCGGTGGTCGCCGTATCATT-3**

**3-GTTCGTCTTCTGCCGTATGCTCTAGCGAGTCAAGGACAGGCGCCTTCGTCACCATAGTTGCGTCTCATGAAGGACACTCGTCGATGACGAGCCTGAATAGCTCATGGGACCGACTTAATCAGAGCACCCGAGCCTCTACACATATTCTCTGTCXXXXXXXXXXXX...XXXXXXXXXXXXGACAGAGAATATGTGTAGACTGCGACGGCTGCTGCGAGGTGCTCACATCTAGAGCCACCAGCGGCATAGTAA-5**

**Sequencing**

**Sequencing product (single ended, with custom primers, deprecated manner of sequencing)**

**I7 read (HyDrop custom primer) R1**

**<--GACAGGCGCCTTCGTCACCATAGTTGCGTCTCATG <--GACAGAGAATATGTGTAGACTGCGACGGCTGCT**

**5-CAAGCAGAAGACGGCATACGAGATCGCTCAGTTCCTGTCCGCGGAAGCAGTGGTATCAACGCAGAGTACTTCCTGTGAGCAGCTACTGCTCGGACTTATCGAGTACCCTGGCTGAATTAGTCTCGTGGGCTCGGAGATGTGTATAAGAGACAGXXXXXXXXXXXX...XXXXXXXXXXXXCTGTCTCTTATACACATCTGACGCTGCCGACGACGCTCCACGAGTGTAGATCTCGGTGGTCGCCGTATCATT-3**

**3-GTTCGTCTTCTGCCGTATGCTCTAGCGAGTCAAGGACAGGCGCCTTCGTCACCATAGTTGCGTCTCATGAAGGACACTCGTCGATGACGAGCCTGAATAGCTCATGGGACCGACTTAATCAGAGCACCCGAGCCTCTACACATATTCTCTGTCXXXXXXXXXXXX...XXXXXXXXXXXXGACAGAGAATATGTGTAGACTGCGACGGCTGCTGCGAGGTGCTCACATCTAGAGCCACCAGCGGCATAGTAA-5**

**CTGTCCGCGGAAGCAGTGGTATCAACGCAGAGTAC--> CTGTCTCTTATACACATCTGACGCTGCCGACGA-->**

**R2 (HyDrop custom primer) I5 read (or via graft)**

**Sequencing product (dual ended, standard primers, used in paper BUT will require custom recipe on some sequencing machines for long index!)**

**I7 read R1**

**<--CAGAGCACCCGAGCCTCTACACATATTCTCTGTC <--GACAGAGAATATGTGTAGACTGCGACGGCTGCT**

**5-CAAGCAGAAGACGGCATACGAGATCGCTCAGTTCCTGTCCGCGGAAGCAGTGGTATCAACGCAGAGTACTTCCTGTGAGCAGCTACTGCTCGGACTTATCGAGTACCCTGGCTGAATTAGTCTCGTGGGCTCGGAGATGTGTATAAGAGACAGXXXXXXXXXXXX...XXXXXXXXXXXXCTGTCTCTTATACACATCTGACGCTGCCGACGACGCTCCACGAGTGTAGATCTCGGTGGTCGCCGTATCATT-3**

**3-GTTCGTCTTCTGCCGTATGCTCTAGCGAGTCAAGGACAGGCGCCTTCGTCACCATAGTTGCGTCTCATGAAGGACACTCGTCGATGACGAGCCTGAATAGCTCATGGGACCGACTTAATCAGAGCACCCGAGCCTCTACACATATTCTCTGTCXXXXXXXXXXXX...XXXXXXXXXXXXGACAGAGAATATGTGTAGACTGCGACGGCTGCTGCGAGGTGCTCACATCTAGAGCCACCAGCGGCATAGTAA-5**

**GTCTCGTGGGCTCGGAGATGTGTATAAGAGACAG--> CTGTCTCTTATACACATCTGACGCTGCCGACGA-->**

**R2 I5 read (or via graft)**
