# Supplementary material for: Hydrop enables droplet-based single-cell ATAC-seq and single-cell RNA-seq using dissolvable hydrogel beads
Source: eLife. 2022 Feb 23;11:e73971. doi: 10.7554/eLife.73971 (PMC8993220; doi:10.7554/eLife.73971)
Supplement: Supplementary file 3. [file elife-73971-supp3.docx]

**Supplementary file to “HyDrop enables droplet based single-cell ATAC-seq and single-cell RNA-seq using dissolvable hydrogel beads”**

Florian V. De Rop^1,2^, Joy N. Ismail^1,2^, Carmen Bravo González-Blas^1,2^, Gert J. Hulselmans^1,2^, Christopher C. Flerin^1,2,4^, Jasper Janssens^1,2^, Koen Theunis^1,2,4^, Valerie M. Christiaens^1,2^, Jasper Wouters^1,2^, Gabriele Marcassa^1,3^, Joris de Wit^1,3^, Suresh Poovathingal^1,#^, and Stein Aerts^1,2,#^

^1^ VIB-KU Leuven Center for Brain & Disease Research

^2^ Laboratory of Computational Biology, Department of Human Genetics, KU Leuven

^3^ Laboratory of Synapse Biology, Department of Neurosciences, KU Leuven

^4^ Aligning Science Across Parkinson’s (ASAP) Collaborative Research Network, Chevy Chase, MD,= 20815.

^#^ Shared last author; correspondence to [suresh.poovathingal@kuleuven.be](mailto:suresh.poovathingal@kuleuven.be) and [stein.aerts@kuleuven.be](mailto:stein.aerts@kuleuven.be).

**HyDrop-RNA**

**Reaction in Emulsion**

**Lysis, mRNA anneals to poly-T** barcodes

5-TTTTTTTTAATACGACTCACTATAGGGAAGCAGTGGTATCAACGCAGAGTAC-[BC1]-CAGCTACTGC-[BC2]-CGAGTACCCT-[BC3]-NNNNNNNNNNTTTTTTTTTTTTTTTTTTTTTTTTT

3-AAAAAAAAAAAAAAAAAA-[mRNA]

**RT with barcoded RT primer (First strand synthesis)**

5-TTTTTTTTAATACGACTCACTATAGGGAAGCAGTGGTATCAACGCAGAGTAC-[BC1]-CAGCTACTGC-[BC2]-CGAGTACCCT-[BC3]-NNNNNNNNNNTTTTTTTTTTTTTTTTTTTTTTTTT-[cDNA]-CCC

3-AAAAAAAAAAAAAAAAAA-[mRNA]

**And template switching with TSO**

5-TTTTTTTTAATACGACTCACTATAGGGAAGCAGTGGTATCAACGCAGAGTAC-[BC1]-CAGCTACTGC-[BC2]-CGAGTACCCT-[BC3]-NNNNNNNNNNTTTTTTTTTTTTTTTTTTTTTTTTT-[cDNA]-CCCATTCACTCTGCGTTGATACCACTGCTT-3

AAAAAAAAAAAAAAAAAAAAAAAAA-[mRNA]-GGGTAAGTGAGACGCAACTATGGTGACGAA-5

**cDNA amplification in bulk (post emulsion breakage)**

**Amplification with TSO-Primer**

TSO-P

5-AAGCAGTGGTATCAACGCAGAGT**-3** -->

5-TTTTTTTTAATACGACTCACTATAGGGAAGCAGTGGTATCAACGCAGAGTAC-[BC1]-CAGCTACTGC-[BC2]-CGAGTACCCT-[BC3]-NNNNNNNNNNTTTTTTTTTTTTTTTTTTTTTTTTT-[cDNA]-CCCATTCACTCTGCGTTGATACCACTGCTT-3

3-AAAAAAAATTATGCTGAGTGATATCCCTTCGTCACCATAGTTGCGTCTCATG-[BC1]-GTCGATGACG-[BC2]-GCTCATGGGA-[BC3]-NNNNNNNNNNAAAAAAAAAAAAAAAAAAAAAAAAA-[cDNA]-GGGTAAGTGAGACGCAACTATGGTGACGAA-5

<--3-TGAGACGCAACTATGGTGACGAA-5

TSO-P

5-AAGCAGTGGTATCAACGCAGAGTAC-[BC1]-CAGCTACTGC-[BC2]-CGAGTACCCT-[BC3]-NNNNNNNNNNTTTTTTTTTTTTTTTTTTTTTTTTT-[cDNA]-CCCATTCACTCTGCGTTGATACCACTGCTT-3

3-TTCGTCACCATAGTTGCGTCTCATG-[BC1]-GTCGATGACG-[BC2]-GCTCATGGGA-[BC3]-NNNNNNNNNNAAAAAAAAAAAAAAAAAAAAAAAAA-[cDNA]-GGGTAAGTGAGACGCAACTATGGTGACGAA-5

**Sequencing library preperation**

**Then, perform NEBNext fragmentation, and dA-tailing**

5-AAGCAGTGGTATCAACGCAGAGTAC-[BC1]-CAGCTACTGC-[BC2]-CGAGTACCCT-[BC3]-NNNNNNNNNNTTTTTTTTTTTTTTTTTTTTTTTTT-[cDNA]-A-3

3-TTCGTCACCATAGTTGCGTCTCATG-[BC1]-GTCGATGACG-[BC2]-GCTCATGGGA-[BC3]-NNNNNNNNNNAAAAAAAAAAAAAAAAAAAAAAAAA-[cDNA]-5

**Then, NEBNext Ligation with Illumina adapter**

5-AAGCAGTGGTATCAACGCAGAGTAC-[BC1]-CAGCTACTGC-[BC2]-CGAGTACCCT-[BC3]-NNNNNNNNNNTTTTTTTTTTTTTTTTTTTTTTTTT-[cDNA]-AGATCGGAAGAGCACACGTCTGAACTCCAGTC\

||||||||||||||||||||||||| ||| |||||||||| ||| |||||||||| ||| |||||||||| |||||||||||||||||||||||| ||||| ||||||||||||| U

3-TTCGTCACCATAGTTGCGTCTCATG-[BC1]-GTCGATGACG-[BC2]-GCTCATGGGA-[BC3]-NNNNNNNNNNAAAAAAAAAAAAAAAAAAAAAAAAA-[cDNA]-TCTAGCCTTCTCGCAGCACATCCCTTTCTCACA/

**And NEB USER enzyme treatment, followed by PCR with P5 index**

I7 index

5-CAAGCAGAAGACGGCATACGAGAT-[I7]-CTGTCCGCGGAAGCAGTGGTATCAACGCAGAGTAC-3

5-AAGCAGTGGTATCAACGCAGAGTAC-[BC1]-CAGCTACTGC-[BC2]-CGAGTACCCT-[BC3]-NNNNNNNNNNTTTTTTTTTTTTTTTTTTTTTTTTT-[cDNA]-AGATCGGAAGAGCACACGTCTGAACTCCAGTC-3

||||||||||||||||||||||||| ||| |||||||||| ||| |||||||||| ||| |||||||||| |||||||||||||||||||||||| ||||| |||||||||||||

3-TTCGTCACCATAGTTGCGTCTCATG-[BC1]-GTCGATGACG-[BC2]-GCTCATGGGA-[BC3]-NNNNNNNNNNAAAAAAAAAAAAAAAAAAAAAAAAA-[cDNA]-TCTAGCCTTCTCGCAGCACATCCCTTTCTCACA-5

<-TCGCAGCACATCCCTTTCTCACA-[i5]-CACATCTAGAGCCACCAGCGGCATAGTAA**-**5

i5 index

5-CAAGCAGAAGACGGCATACGAGAT-[i7]-CTGTCCGCGGAAGCAGTGGTATCAACGCAGAGTAC-[BC1]-CAGCTACTGC-[BC2]-CGAGTACCCT-[BC3]-NNNNNNNNNNTTTTTTTTTTTTTTTTTTTTTTTTT-[cDNA]-AGATCGGAAGAGCGTCGTGTAGGGAAAGAGTGT-[i5]-GTGTAGATCTCGGTGGTCGCCGTATCATT-3

3-GTTCGTCTTCTGCCGTATGCTCTA-[i7]-GACAGGCGCCTTCGTCACCATAGTTGCGTCTCATG-[BC1]-GTCGATGACG-[BC2]-GCTCATGGGA-[BC3]-NNNNNNNNNNAAAAAAAAAAAAAAAAAAAAAAAAA-[cDNA]-TCTAGCCTTCTCGCAGCACATCCCTTTCTCACA-[i5]-CACATCTAGAGCCACCAGCGGCATAGTAA-5

**Sequencing**

HyDrop_CustSeq_Short TruSeq Read 1

<--3-GACAGGCGCCTTCGTCACCATAGTTGCGTCTCATG-5 <--3-TCTAGCCTTCTCGCAGCACATCCCTTTCTCACA-5

5-CAAGCAGAAGACGGCATACGAGAT-[i7]-CTGTCCGCGGAAGCAGTGGTATCAACGCAGAGTAC-[BC1]-CAGCTACTGC-[BC2]-CGAGTACCCT-[BC3]-NNNNNNNNNNTTTTTTTTTTTTTTTTTTTTTTTTT-[cDNA]-AGATCGGAAGAGCGTCGTGTAGGGAAAGAGTGT-[i5]-GTGTAGATCTCGGTGGTCGCCGTATCATT-3

3-GTTCGTCTTCTGCCGTATGCTCTA-[i7]-GACAGGCGCCTTCGTCACCATAGTTGCGTCTCATG-[BC1]-GTCGATGACG-[BC2]-GCTCATGGGA-[BC3]-NNNNNNNNNNAAAAAAAAAAAAAAAAAAAAAAAAA-[cDNA]-TCTAGCCTTCTCGCAGCACATCCCTTTCTCACA-[i5]-CACATCTAGAGCCACCAGCGGCATAGTAA-5

5-CTGTCCGCGGAAGCAGTGGTATCAACGCAGAGTAC-3--> 5-AGATCGGAAGAGCGTCGTGTAGGGAAAGAGTGT-3-->

HyDrop_CustSeq_R2 TruSeq Index 2
